# Fetal adverse effects following NSAID or metamizole exposure in the 2nd and 3rd trimester: an evaluation of the German Embryotox cohort

**DOI:** 10.1186/s12884-022-04986-4

**Published:** 2022-08-26

**Authors:** Katarina Dathe, Johanna Frank, Stephanie Padberg, Stefanie Hultzsch, Evelin Beck, Christof Schaefer

**Affiliations:** Charité – Universitätsmedizin Berlin, corporate member of Freie Universität Berlin, Humboldt-Universität zu Berlin, and Berlin Institute of Health, Institut für Klinische Pharmakologie und Toxikologie, Pharmakovigilanz- und Beratungszentrum für Embryonaltoxikologie (Embryotox), Augustenburger Platz 1, 13353 Berlin, Germany

**Keywords:** Anti-inflammatory agents, non-steroidal [Mesh], Pregnancy trimester, second [MeSH], Pregnancy trimester, third [MeSH], Fetus [Mesh], Ductus arteriosus [MeSH], Closure of ductus arteriosus Botalli, Stillbirth [Mesh], Oligohydramnios [MeSH], Renal insufficiency [MeSH], Persistent fetal circulation syndrome [Mesh], Ductus arteriosus, patent [MeSH]

## Abstract

**Background:**

Non-steroidal anti-inflammatory drugs (NSAID) are frequently used to treat pain, fever and inflammatory conditions. Due to evidenced fetotoxicity, treatment with NSAID and metamizole should be avoided in the 3rd trimester of pregnancy. There is an ongoing debate on fetotoxic risk of 2nd trimester use which is why we have conducted this study.

**Methods:**

In this observational cohort study outcome of pregnancies with NSAID and/or metamizole exposure in the 2nd and/or 3rd trimester (study cohort *n* = 1092) was compared with pregnancies exposed to NSAID and/or metamizole in the 1st trimester only (comparison cohort, *n* = 1154). The WHO-UMC system was used to assess causality between study medication and study endpoints. Prenatal study endpoints were constriction of ductus arteriosus Botalli, oligohydramnios, late spontaneous abortion (SAB) or stillbirth. Postnatal study endpoints were patent ductus arteriosus (PDA), anomalies of the right heart ventricle, primary pulmonary hypertension (PPHT), and neonatal impairment of kidney function.

**Results:**

Ductus arteriosus constriction was diagnosed in 5/1092 (0.5%) in the study cohort versus 0/1154 pregnancies in the comparison cohort. In one fetus, ductus arteriosus constriction and oligohydramnios occurred already in the late 2nd trimester after long-term NSAID exposure. Oligohydramnios was diagnosed in 41/1092 (3.8%) in the study cohort versus 29/1154 (2.5%) cases in the comparison cohort [RR, 1.5 (95% CI 0.9–2.4)]. Limited to 2nd trimester, oligohydramnios occurred in 8/904 (0.9%) versus 2/1154 (0.2%) pregnancies [RR, 5.1 (95% CI 1.1–24.0)]. At least in four of the 2nd trimester exposed pregnancies NSAID exposure lasted several weeks. Late SAB or stillbirth occurred in 14/1092 (1.3%) versus 17/1154 (1.5%). Postnatal cardiovascular or renal pathology did not differ between the cohorts.

**Conclusions:**

NSAID use in the 2nd trimester limited to a few days does not appear to pose a relevant risk. Use for longer periods in the advanced 2nd trimester, however, may cause oligohydramnios and ductus arteriosus constriction similar to effects observed after 3rd trimester use.

**Supplementary Information:**

The online version contains supplementary material available at 10.1186/s12884-022-04986-4.

## Background

Pain, fever and inflammatory conditions are not uncommon during pregnancy [1, 2]. If untreated, they may impair the health of both mother and child [3–5]. Therefore, in addition to non-drug options, adequate medication is often required. More than 50% of pregnant women take over-the-counter analgesics such as non-steroidal anti-inflammatory drugs (NSAID) or paracetamol [6, 7].

Due to their well-known fetotoxic risks, NSAID and metamizole should not be used during the 3rd trimester [8–12]. In addition, there are several reports describing fetal adverse effects after NSAID therapy in the advanced 2nd trimester [13]. However, the magnitude of this risk has not been sufficiently investigated yet. There are many open questions, e.g. from which gestational week (GW) and duration of exposure the risk increases and whether certain NSAID carry a higher risk.

The FDA recommends avoiding the use of NSAID at GW 20 or later with the exception of low-dose-aspirin [14]. This recommendation is based on the fact that exposure to NSAID can decrease amniotic fluid. However, drug specificity including dose, duration and treatment indication need to be taken into account.

Due to their frequent use, further studies on the risk of these drugs are urgently required. The objective of this study was, therefore, to assess the risk of defined fetal adverse effects and pregnancy outcome after exposure to NSAID and/or metamizole in the 2nd and 3rd trimester, with a specific focus on 2nd trimester exposure.

## Methods

### Data collection

The Embryotox Center of Clinical Teratology and Drug Safety in Pregnancy (Embryotox), Berlin, offers counselling and risk assessment on drug use in pregnancy to health care professionals (HCP) and patients [15]. In addition, Embryotox serves as a national clearinghouse for suspected adverse drug reactions in pregnancy.

Upon contact to Embryotox, details on all drug exposures (duration of treatment, dosage, ATC-codes), treatment indications (MedDRA) and maternal medical history are recorded. Approximately 8 weeks after the expected date of delivery, follow-up data are obtained by a standardized procedure using a questionnaire resulting in a response rate of approx. 75%. Details on further drug exposures, complications during pregnancy, delivery and neonatal outcome including congenital anomalies are asked for. In cases of incomplete or inconsistent response, the patient and/or her HCP are contacted for further information and medical records. Following a case by case plausibility check data are archived in the Embryotox database (VigilanceOne, PharmApp Solutions GmbH).

### Study design and study cohorts

Only cases with a prospective pregnancy enrolment were included in this cohort study. ‘Prospective’ in this context means that there was an ongoing pregnancy at the first contact to Embryotox and neither the pregnancy outcome nor pathological findings from prenatal examinations were known. All requests handled by Embryotox between 01/01/2008 and 31/12/2017 (estimated date of birth) with completed follow-up and exposure to the study medication were considered. The Ethics Committee of the Charité - Universitätsmedizin Berlin, Germany (ref. EA2/129/18, 14 August 2018) has approved this study and has waived the requirement of a separate informed study consent.

The study medication included: NSAID, acetylsalicylic acid (ASA) (> 300 mg/d, analgesic dosage) and metamizole. For a detailed list of substances that have been included see Additional Table S1.

Study and comparison cohort were defined as following:

- The study cohort comprised pregnancies with systemic exposure to the study medication at any time in the 2nd and/or 3rd trimester. Treatment may have started before or during the 2nd/3rd trimester.
- The comparison cohort included pregnancies that were systemically exposed to the study medication in the 1st trimester only. Treatment may have started before or during the 1st trimester.

Intake of study medication had to be clearly assignable to pregnancy trimesters (1st trimester: GW 2 + 0 to GW 12 + 6; 2nd trimester: GW 13 + 0 to 26 + 6; 3rd trimester: GW 27 + 0 to delivery). Cases were excluded if assignment was not possible (e.g. ‘sometimes in pregnancy’, ‘if necessary’, ‘as needed’). GW were calculated either by ultrasound determination in early pregnancy or the first day of the last menstrual period (LMP) if ultrasound information was not available. Pregnancies with embryotoxic or fetotoxic co-medication were not excluded, but these co-medications were taken into account for case-by-case assessment.

The main focus of this study was on fetal effects after exposure to the study medication in the 2nd and/or 3rd trimester. Therefore, pregnancies ending before GW 13 + 0 [spontaneous abortions (SAB) and elective terminations of pregnancies (ETOP)] were excluded from both cohorts. Twin pregnancies with a SAB of one embryo in the 1st trimester were included.

**Fig. 1:**
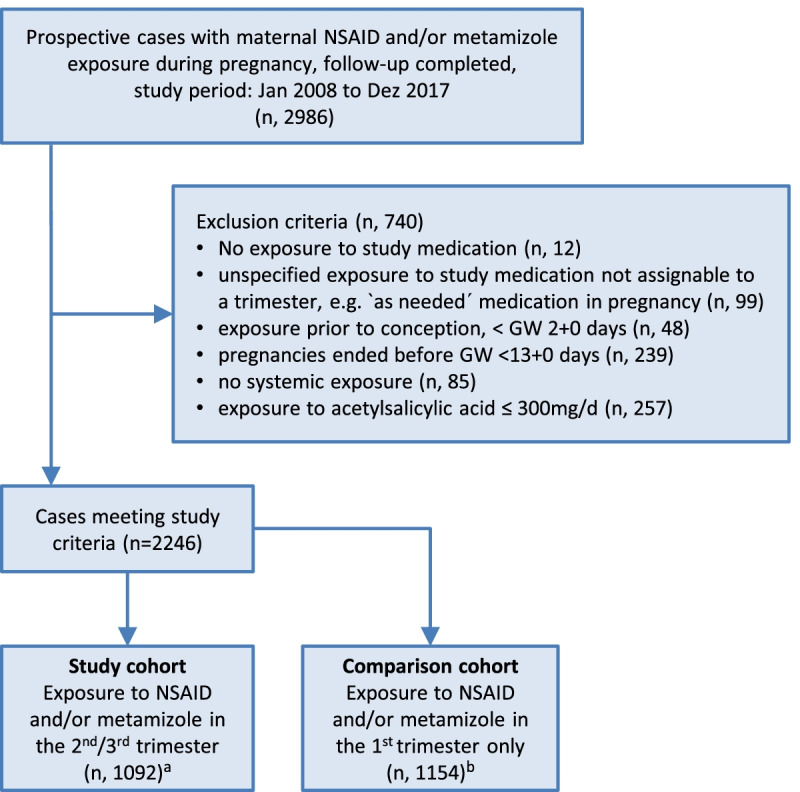
Flow chart. Flow chart for generating the study cohort and the comparison cohort. ^a^ The study cohort includes 31 twin and one triplet pregnancy. 1098 infants were live-born. ^b^ The comparison cohort included 14 twin pregnancies. 1133 infants were liveborn. Further information on pregnancy outcomes in both cohorts are given in the result section

The flow chart briefly shows the selection criteria to generate the study cohorts (Fig. 1).

### Study endpoints

Prenatal study endpoints:

- intrauterine constriction or closure of ductus arteriosus Botalli (possibly associated fetal heart findings as tricuspid insufficiency and right ventricular overload were also recorded),
- reduced amniotic fluid volume, oligohydramnios, anhydramnios,
- spontaneous abortion after the 1st trimester and stillbirth.

Causality assessment was conditional to a study drug exposure preceding the diagnosis of the prenatal study end point.

Postnatal study endpoints:

- patent fetal ductus arteriosus Botalli (PDA) in the newborn,
- cardiac findings indicative for right ventricular strain such as tricuspid insufficiency, right ventricular dilatation and/or hypertrophy, primary pulmonary hypertension (PPHT),
- (functional) renal disorders and renal insufficiency.

For causality assessment between the exposure to study medication and prenatal study endpoints, the World Health Organization Uppsala Monitoring Centre (WHO-UMC) system for standardized case causality assessment was used [16]. The assessment was carried out independently by two experts. In cases of inconsistency, a third expert was involved for the final decision.

### Data analysis

For the comparison of maternal and infant characteristics descriptive statistics was applied. Relative risks of the defined prenatal study endpoints were calculated by dividing the number of affected pregnancies by all pregnancies in each cohort. For postnatal study endpoints, relative risks were calculated by dividing the affected infants by all live-born infants in each cohort. Risk ratios (RR) and 95% confidence intervals (CI) were calculated for dichotomous endpoints. Risk ratios were calculated by dividing the relative risk in the study cohort by the relative risk in the comparison cohort. No risk ratios are presented for the time-dependent endpoints pregnancy loss and ETOP because risk ratios do not account for delayed study entry and competing risks. Analyses were performed using R version 3.3 (R Development Core Team, Vienna, Austria).

### Participant and public involvement

There was no patient or public involvement in setting the research questions, designing the study or interpretation of study results.

### Funding

This work was supported by the German Federal Institute for Drugs and Medical Devices (BfArM). The funder had no role in study design, data collection and analysis, decision to publish, or preparation of the manuscript. The study is listed in the German Clinical Trials Register under DRKS00015617. Open Access funding enabled and organized by Projekt DEAL.

## Results

The study cohort comprised *n* = 1092 pregnancies with an exposure to NSAID/metamizole in the 2nd and/or 3rd trimester resulting in *n* = 1098 live-born infants. This cohort included 31 twin pregnancies and one triplet. The comparison cohort consisted of *n* = 1154 pregnancies (including 14 twin pregnancies) exposed to NSAID/metamizole only in the 1st trimester and resulted in 1133 live-born infants. See also flow chart Fig. 1.

### Cohort characteristics

#### Maternal and neonatal characteristics

With a few exceptions maternal characteristics were similar in both cohorts (Table 1). Smoking and alcohol consumption were less prevalent in the study cohort (nicotine: 15.3% vs. 20.5%, alcohol: 12% vs. 15.6%). The proportion of women with a previous SAB was slightly higher in the study cohort than in the comparison cohort (19.5% vs. 13.4%) as was the proportion of women who wished to become pregnant (96.1% vs. 89.6%).

**Table 1:** Maternal characteristics of the study cohorts

Cohorts	NSAID	Comparison
**GW at first contact, n**	1092	1154
Median (IQR)	12.4 (7.7–20.9)	7.7 (6–10.1)
**Age (years), n**	1091	1154
Median (IQR), (min-max)	33 (30–36) (15–49)	32 (28–35) (17–45)
**Pre-pregnancy BMI, n**	1061	1095
Median, (kg/m^2^), (IQR)	23.1 (20.9–26.7)	22.8 (20.7–26.2)
**Educational level, n**	779	704
≤ 9 years, n (%)	4 (0.5)	8 (1.1)
> 9 and ≤ 13.5 years, n (%)	209 (26.8)	229 (32.5)
Acadamic degree, n (%)	342 (43.9)	285 (40.5)
**Smoking, n**	1089	1144
No, n (%)	922 (84.7)	910 (79.5)
≤ 5 cig/day, n (%)	45 (4.1)	64 (5.6)
> 5 cig/day, n (%)	122 (11.2)	170 (14.9)
**Alcohol, n**	1089	1142
No, n (%)	959 (88.1)	964 (84.4)
≤ 1 drink/day, n (%)	89 (8.2)	107 (9.4)
> 1 drink/day, n (%)	41 (3.8)	71 (6.2)
**Pregnancy wanted, n**	863	963
Yes, n (%)	829 (96.1)	863 (89.6)
Indifferent, n (%)	23 (2.7)	79 (8.2)
No, n (%)	11 (1.3)	21 (2.2)
**Previous pregnancies, n**	1089	1149
0, n (%)	430 (39.5)	476 (41.4)
1, n (%)	363 (33.3)	366 (31.9)
2, n (%)	161 (14.8)	169 (14.7)
≥ 3, n (%)	135 (12.4)	138 (12)
**Previous deliveries, n**	1089	1147
0, n (%)	530 (48.7)	561 (48.9)
1, n (%)	394 (36.2)	380 (33.1)
2, n (%)	125 (11.5)	139 (12.1)
≥ 3, n (%)	40 (3.7)	67 (5.8)
**Previous spontaneous abortions, n**	1087	1143
0, n (%)	876 (80.6)	989 (86.5)
1, n (%)	143 (13.2)	109 (9.5)
≥ 2, n (%)	68 (6.3)	45 (3.9)
**Previous children with birth defect, n**	1088	1144
0, n (%)	1059 (97.3)	1118 (97.7)
1, n (%)	25 (2.3)	23 (2)
≥ 2, n (%)	4 (0.4)	3 (0.3)

The absolute number of parameters differ due to missing values. *BMI* Body mass index, *GW* Gestational week, *IQR* Interquartile range, *n* Number of cases with available information

Both cohorts also showed similarities in the neonatal characteristics. The average gestational age at birth was GW 39.0 in the study cohort and GW 39.4 GW in the comparison cohort. The proportion of preterm infants was slightly higher in the study cohort (14.8% vs. 10.4%). There were no relevant differences for birth weight, body length, and head circumference (Additional Table S2).

#### Exposure pattern in the 2nd/3rd trimester

The exposure to the study medication was heterogeneous. One third of the study cohort was exposed for less than 7 days (366/1092, 33.5%), whereas 185/1092 (16.9%) used NSAID/metamizole more than 28 days (Additional Table S3). 609/1092 (55.8%) were exclusively exposed in the 2nd trimester, 295/1092 (27.0%) in the 2nd and 3rd trimester and 150/1092 (13.7%) used the study drugs only in the 3rd trimester. 38/1092 were not clearly assignable to the 2nd or 3rd trimester.

Additional Table S1 provides an overview of the study medication by substance in both cohorts. In the 2nd/3rd trimester exposure cohort ibuprofen was most frequently used (76.8%), followed by diclofenac (7.6%) and metamizole (6.1%). In the comparison cohort ibuprofen was used in 50.5%, followed by metamizole (20.1%) and diclofenac (10.4%). 12.1% of patients in the study cohort took more than one NSAID and/or metamizole (Additional Table S4).

Headache was the most frequently reported treatment indication (37.6%) among the 2nd/3rd trimester exposed followed by 23.7% for musculoskeletal pain (back or joint pain) and other pain symptoms (21.0%). Tocolysis was reported in only 1.5%.

### Study endpoints

**Fig. 2:**
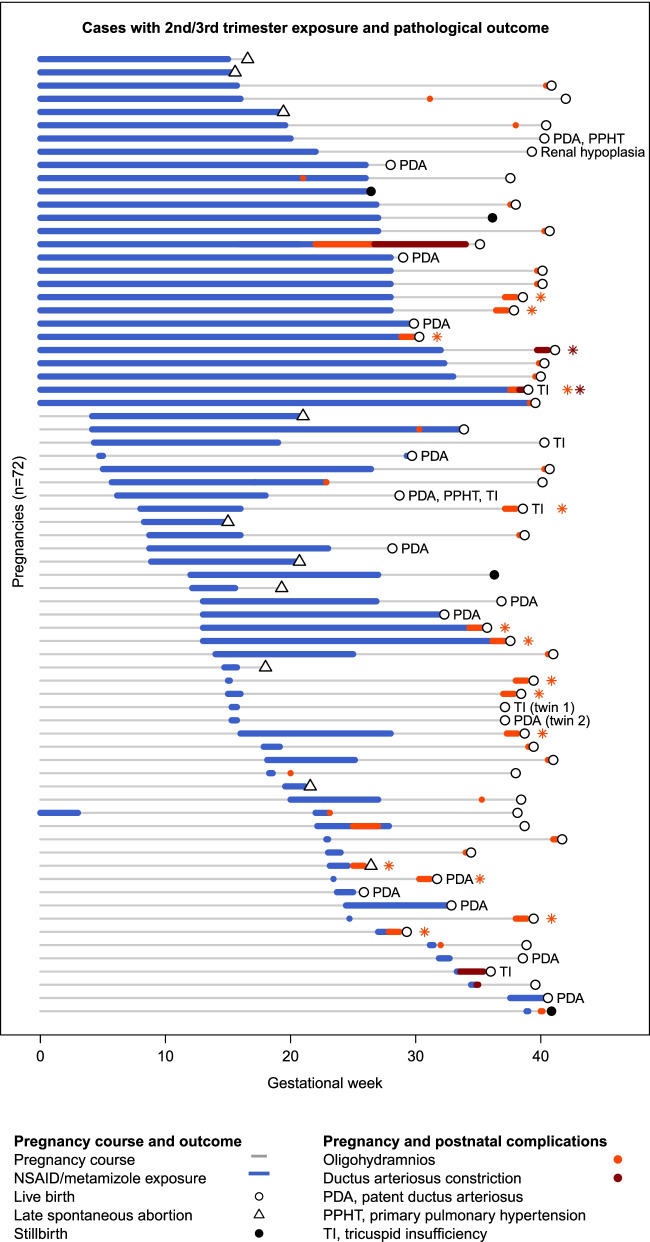
Cases with 2nd/3rd trimester exposure and pathological outcome. Cases are labelled with an asterisk if gestational week and duration of the study endpoint were unknown (red, ductus arteriosus constriction; orange, oligohydramnios). The order (pregnancies, *n* = 72) is determined by the start and duration of exposure to study medication

After NSAID/metamizole exposure in the 2nd or 3rd trimester, 72 cases with study-specific endpoints were identified, see Fig. 2 and Table 2 for an overview and Additional Table S5 with details of the 72 affected cases.

**Table 2:** Frequency of endpoints by trimester of diagnosis

	Study cohort, pregnancies (n, 1092), exposure: 2nd and/or 3rd trimester, n (%)	Comparison cohort, pregnancies (n, 1154), exposure: 1st trimester, n (%)	Risk ratio, RR (95% CI)
**Constriction of ductus arteriosus**	5/1092 (0.5)	0/1154 (0.0)	
•2nd trimester	1/904 (0.1)	0/1154 (0.0)	
•3rd trimester	2/1092 (0.2)	0/1154 (0.0)	
•Not clearly assignable to trimester	2/1092 (0.2)	0/1154 (0.0)	
**Oligohydramnios**	41/1092 (3.8)	29/1154 (2.5)	1.5 (0.9–2.4)
•2nd trimester	8/904 (0.9)	2/1154 (0.2)	5.1 (1.1–24.0)
•3rd trimester	23/1092 (2.1)	20/1154 (1.7)	
•Not clearly assignable to trimester	10/1092 (0.9)	7/1154 (0.6)	
**Late SAB/fetal death**	14/1092 (1.3)	17/1154 (1.5)	
•2nd trimester	11/904 (1.2)	12/1154 (1.0)	
•3rd trimester	3/1092 (0.3)	5/1154 (0.4)	
**ETOP (after 1st trimester)**	10/1092 (0.9)	16/1154 (1.4)	
	**Live-born infants** **(n, 1098)**	**Live-born infants** **(n, 1133)**	
**Patent ductus arteriosus**	15/1098 (1.4)	10/1133 (0.9)	1.6 (0.7–3.4)
•term-born (≥ 37 + 0 GW)	4/933^a^ (0.4)	5/1010^a^ (0.5)	0.9 (0.2–3.2)
•preterm-born (< 37 + 0 GW)	11/162^a^ (6.8)	5/117^a^ (4.3)	1.6 (0.6–4.5)
**PPHT and other cardiac anomalies**^b^	5/1098 (0.5)	2/1133 (0.2)	2.6 (0.5–13.3)
**Renal disorder**	1/1098 (0.1)	1/1133 (0.1)	1.0 (0.1–16.5)

Multiple endpoints in one pregnancy are listed and counted separately. ^a^ Number of live-born infants with information on gestational week (GW) at birth. ^b^ Other cardiac anomalies include tricuspid insufficiency and right ventricular load. Only those pregnancies definitely exposed in the 2nd trimester were considered to calculate the frequency in the 2nd trimester. *ETOP* Elective termination of pregnancy, *PPHT* Primary pulmonary hypertension, *SAB* Spontaneous abortion

#### Intrauterine constriction of the ductus arteriosus Botalli

Intrauterine constriction of ductus arteriosus was diagnosed in 5/1092 cases (0.5%) (Table 2 and Additional Table S5 cases 1–5) among 2nd/3rd trimester exposed pregnancies. In one of these, constriction occurred already in the late 2nd trimester (GW 27) along with the diagnosis of oligohydramnios, after amniotic fluid reduction had already been noted at GW 22 (Additional Table S5 case 4). The symptoms improved after discontinuation of the long-term medication with both, diclofenac (GW 0 to 27) and ibuprofen (GW 16 to 21). In two fetuses, stenosis of ductus arteriosus was diagnosed in the 3rd trimester (Additional Table S5 case 1 and 2) and in two further fetuses the diagnosis of ductus arteriosus constriction could not definitely be assigned to trimester 2 or 3 (Additional Table S5 case 3 and 5), but long-term NSAID use was reported in both cases. In the comparison cohort (*n* = 1154), there was no case of intrauterine constriction of ductus arteriosus.

#### Oligohydramnios

Oligo- or anhydramnios was diagnosed in 41/1092 pregnancies (3.8%) of the study cohort versus 29/1154 (2.5%) in the comparison cohort [RR 1.5 (95% CI 0.9–2.4)] (Table 2).

Limited to 2nd trimester diagnosis of oligo−/anhydramnios, 8/904 2nd trimester exposed cases (0.9%; additional Table S5 cases 4, 6–10, 42, 43) were affected versus 2/1154 (0.2%) of the comparison cohort [RR 5.1 (95% CI 1.1–24.0)]. At least in four of the 2nd trimester exposed cases (cases 4, 6, 8, 9) NSAID exposure lasted several weeks.

In 10/1092 cases (0.9%) versus 7/1154 (0.6%), respectively, the GW of onset of oligohydramnios could not be specified.

#### Pregnancy losses

Among 2nd or 3rd trimester exposed pregnancies 14/1092 (1.3%) resulted in SAB or stillbirth (Table 2). Of these, 11 were observed in the 2nd and three in the 3rd trimester. In all cases, a causal relationship between NSAID and outcome was unlikely (Additional Table S5). ETOP was performed in 10 pregnancies, 7 because of fetal malformations or chromosomal disorders and three ETOPs for non-medical reasons. In the comparison cohort, 17/1154 (1.5%) pregnancies resulted in SAB or stillbirth and 16/1154 were terminated. In 9 of these fetal malformations or chromosomal disorders were diagnosed.

#### Patent ductus arteriosus (PDA)

Among 2nd or 3rd trimester exposed pregnancies 15/1098 (1.4%) live-born infants were diagnosed with PDA (Table 2). As expected, the prevalence for PDA was higher (11/162; 6.8%) in the subgroup of preterm infants (< 37 + 0 GW) (Additional Table S5, cases 42, 62–71), including one infant (case 65) with additional tricuspid insufficiency and PPHT. Exposure to NSAID for several weeks was reported in 8/11 preterm born infants (Additional Table S5, cases 62–65, 67, 68, 70, 71) and to metamizole in 1/11 (case 66). Limited to mature born infants, 4/933 (0.4%) had PDA (Additional Table S5 cases 58–61).

In the comparison cohort, the overall rate of PDA was 10/1133 (0.9%) including 5/117 (4.3%) in preterm infants and 5/1010 (0.5%) in mature born infants.

#### Cardiac findings indicative for right ventricular strain and PPHT

Tricuspid insufficiency and PPHT without ductus arteriosus constriction was present in 5/1098 (0.5%) in the study cohort and in 2/1133 (0.2%) in the comparison cohort.

#### Postnatal renal disorders

In both cohorts, one child was reported with unilateral renal hypoplasia with ibuprofen use `as needed´ until GW 22 in one case and until GW 11 + 6 in the other case.

## Discussion

This study is one of the largest to date on the safety of NSAID and metamizole after the 1st trimester of pregnancy, with a focus on fetal adverse effects resulting from 2nd trimester exposure. Our findings confirm that long-term medication can lead to oligohydramnios and ductus arteriosus constriction in the advanced 2nd trimester whereas short-term use in the 2nd trimester does not seem to be associated with such a risk.

One of the strengths of the evaluated Embryotox database on which these study cohorts are based is the large number of well documented pregnancies including – in contrast to many other study evaluations – both over the counter (OTC) and prescription medication. The Embryotox consultation service is available to all attending physicians and patients free of charge, regardless of e.g. their socio-economic status, medical knowledge, or ethnic group. Case by case plausibility checks of exposure and outcome data are performed by experienced physicians and medical documentarists – see detailed description of procedures published elsewhere [15]. Nevertheless, even if the pregnancy follow-up-data used for this study were collected with care and thoroughness, we cannot completely rule out underreporting of adverse events by patients and/or their HCP. As a further limitation, the Embryotox cohort may not reflect the average population of pregnant woman in Germany. Embryotox users tend have a higher level of education which may constitute a low risk population [17, 18]. This could represent a selection bias. However, study and comparison pregnancies for this study evaluation were taken from the same data pool and demonstrated similar characteristics. Using the same medication earlier in pregnancy as comparison favours a comparable spectrum of underlying indications in both cohorts. In the study cohort, the median GW at first contact to Embryotox was later (GW 12 + 3) than in the comparison cohort (GW 7 + 5), which appears plausible due to the earlier trimester of exposure in the comparison cohort. This circumstance would probably affect results of 1st trimester embryotoxicity such as early abortion rate. However, early adverse events do not belong to our study goals.

In our study, there was only one fetus with confirmed intrauterine ductus constriction and oligohydramnios in the late 2nd trimester (Additional Table S5 case 4). Causality with the daily exposure to diclofenac and intermittent co-medication with ibuprofen from GW 16 to GW 21 and the findings seems plausible due to the temporal relationship and recovery after discontinuation of the medication and therefore were assessed as `certain´ according to the WHO-UMC classification. The subsequent daily intake of paracetamol did not appear to have negative effects. A literature review identified case reports of a total of 33 fetuses presenting intrauterine ductal constriction in the 2nd trimester after NSAID exposure with the earliest findings at GW 25. However, in most of these cases indometacin was used for tocolysis [13].

Two of the other 4 cases with ductus arteriosus stenosis in our cohort (Additional Table S5 cases 1 and 2) showed a prenatal normalization of findings after discontinuation of NSAID medication. In the remaining two cases normal cardiac function after birth was confirmed in one (Additional Table S5 case 3) and ventricular hypertrophy and tricuspid insufficiency was diagnosed in another one (Additional Table S5 case 5).

Prenatal stenosis of the ductus arteriosus may occur spontaneously, a definite prevalence is not available. A case series by Leal et al. described 5 fetuses with occlusion of the ductus arteriosus, 3 of which occurred in the absence of maternal medication and two in association with maternal indometacin intake [19]. Luchese et al. observed 20 fetuses with prenatal ductal stenosis in a cohort of 7000 pregnancies, of which 13/7000 (0.2%) were classified as `idiopathic´. In the remaining 7 cases, maternal exposure to indometacin, diclofenac, or ASA preceded ductal constriction [20]. In a case series, Enzensberger et al. presented 3 cases with idiopathic ductal stenosis and favourable outcome in all newborns [21]. In another study based on echocardiography findings, 45 fetuses with ductal stenosis/occlusion between 27 and 38 weeks of gestation were identified among 26000 pregnant women. Twenty-nine cases were found to be related to prior NSAID exposure, of which 8 patients had taken only a single dose. Furthermore, 8/26000 (0.03%) cases without any maternal medication were classified as `idiopathic´. An additional 8 patients were assigned to a third group who had taken medications other than NSAID in temporal relation to the onset of ductal stenosis [8]. There is an ongoing debate on further risk factors favouring ductal stenosis such as other medications and food ingredients [8, 22–24].

All in all, the risk for intrauterine ductus stenosis after NSAID exposure in the 2nd trimester seems to be very low. However, some findings may remain undetected because echocardiography of the ductus arteriosus is not routinely performed after NSAID exposure in the 2nd trimester and, consequently, the incidence may be underestimated.

PPHT is associated with high morbidity and mortality [25, 26]. Intrauterine exposure to NSAID is discussed as a risk factor for developing PPHT [27–31]. Two children in our study cohort were diagnosed with PPHT. One infant (Additional Table S5 case 65) had pulmonary hypoplasia, probably caused by prolonged anhydramnios after premature rupture of membranes. Tricuspid insufficiency and right ventricular hypertrophy in this child were probably a consequence of pulmonary hypertension and right ventricular pressure increase. A causal relationship with the study medication up to GW 18 seems unlikely. The second child (Additional Table S5 case 59) was born with a cystic adenomatoid malformation of the lung, which more likely caused PPHT than the ibuprofen exposure up to GW 20.

Tricuspid insufficiency may occur with intrauterine ductal stenosis as a result of increased pulmonary pressure and right heart pressure. The number of infants with tricuspid insufficiency without ductal stenosis was slightly higher in the study cohort than in the comparison cohort (3/1092, 0.3% versus 1/1154, 0.1%). However, maternal NSAID medication did not persist beyond GW 19 (Additional Table S5 cases 41, 57, 58). Isolated tricuspid insufficiency in the fetus may occur transiently and without clinical significance [32].

PDA was more often diagnosed among preterm infants exposed to NSAID in the 2nd or 3rd trimester compared to preterm infants exclusively exposed in the 1st trimester (6.8% versus 4.3%). This was particularly evident in infants with a birth weight below 1500 g (28.0% versus 18.8%, data not shown). There was no relevant difference for mature infants (0.4% versus 0.5%). It is well known that the incidence of PDA depends on gestational age at birth, birth weight, and neonatal morbidity [33, 34]. The incidence of PDA in preterm infants in the cohort with 2nd/3rd trimester exposure is comparable to the frequency reported in the literature [35, 36].

We observed a slight increase of oligohydramnios (3.8% versus 2.5%) in the 2nd/3rd trimester exposed study cohort. Among other causes [37] NSAID use in the 3rd trimester is a well-known risk factor for oligohydramnios [38]. Inhibition of prostaglandin synthesis due to NSAID may compromise the renal function of the fetus. There are also reports of oligohydramnios following metamizole use during the 3rd trimester [9, 39]. Limited to 2nd trimester exposure the relative risk for oligohydramnios was stronger than for the combined 2nd/3rd trimester cohort and reached statistical significance [0.9% versus 0.2%, RR 5.1 (95% CI 1.1–24.0)]. Longer treatment intervals during the 2nd trimester compared to the restricted use following established warnings against 3rd trimester use could be an explanation. However, following WHO-UMC causality assessment there were only one `certain´ and two `probable´ relations among 2nd trimester diagnosed oligohydramnios (Additional Table S5 case 4, 6, 9).

Finally, we did not observe an increased risk for late abortions and stillbirths as worst case scenarios of cardiac decompensation following intrauterine occlusion of the ductus arteriosus [40, 41].

## Conclusion

Analgesic or antipyretic NSAID use limited to a few days in the 2nd trimester does not appear to carry a relevant risk. Use for longer periods of time in the advanced 2nd trimester, however, or exposure in the 3rd trimester may cause fetal adverse effects. Close observation to assess ductal flow with Doppler echocardiography, and controlling amniotic fluid volume by ultrasound is advisable after repeated or prolonged NSAID use in late 2nd trimester or beyond.

## Supplementary Information

**Additional file 1: Table S1.** Number of exposures to study medication per substance.

**Additional file 2: Table S2.** Neonatal characteristics.

**Additional file 3: Table S3.** Exposure intervals of study medication (a) and assignment of exposure to trimesters (b).

**Additional file 4: Table S4.** Multiple exposure to study medication.

**Additional file 5: Table S5.** Summary of cases with 2nd/3rd trimester study medication and defined study endpoints.

## Data Availability

Relevant data generated or analysed during this study are included in this published article and its supplementary information files.

## Abbreviations

ATC-code: Anatomical Therapeutic Chemical code
ASA: Acetylsalicylic acid
CI: Convidence interval
ETOP: Elective termination of pregnancy
GW: Gestational week
LMP: Last menstrual period
NSAID: Non-steroidal anti-inflammatory drugs
SAB: Spontaneous abortion
PDA: Patent ductus arteriosus
PPHT: Primary pulmonary hypertension
RR: Risk ratio
MedDRA: Medical Dictionary for Regulatory Activities
WHO-UMC: World Health Organization Uppsala Monitoring Centre
