# Supplementary material for: Fetal adverse effects following NSAID or metamizole exposure in the 2nd and 3rd trimester: an evaluation of the German Embryotox cohort
Source: BMC Pregnancy Childbirth. 2022 Aug 26;22:666. doi: 10.1186/s12884-022-04986-4 (PMC9413886; doi:10.1186/s12884-022-04986-4)
Supplement: Supplementary file 1 — Additional file 1: Table S1. Number of exposures to study medication per substance. [file 12884_2022_4986_MOESM1_ESM.pdf]

Table S1. Number of exposures to study medication per substance.

Substance	Study cohort, exposure: 2nd and/or 3rd trimester (n, 1171[a])	Comparison cohort, exposure: 1st trimester (n, 1408[a])
	n (%)	n (%)
Ibuprofen	899 (76,8)	710 (50,4)
Diclofenac	89 (7,6)	146 (10,4)
Metamizole	72 (6,1)	283 (20,1)
Indometacin	38 (3,2)	3 (0,2)
Acetylsalicylic acid (>300 mg/d)	36 (3,1)	119 (8,5)
Naproxen	16 (1,4)	26 (1,8)
Etoricoxib	6 (0,5)	47 (3,3)
Mefenamic acid	6 (0,5)	4 (0,3)
Celecoxib	2 (0,2)	13 (0,9)
Meloxicam	1 (0,1)	8 (0,6)
Bufexamac	1 (0,1)	1 (0,1)
Propyphenazone	1 (0,1)	3 (0,2)
Acemetacin	1 (0,1)	3 (0,2)
Lornoxicam	1 (0,1)	1 (0,1)
Sulindac	1 (0,1)	0 (0,0)
Ketoprofen	1 (0,1)	0 (0,0)
Dexibuprofen	0 (0,0)	7 (0,5)
Dexketoprofen	0 (0,0)	19 (1,3)
Parecoxib	0 (0,0)	4 (0,3)
Aceclofenac	0 (0,0)	1 (0,1)
Flurbiprofen	0 (0,0)	3 (0,2)
Nimesulide	0 (0,0)	2 (0,1)
Salicylamide	0 (0,0)	1 (0,1)
Etodolac	0 (0,0)	1 (0,1)
Aminophenazone	0 (0,0)	2 (0,1)
Tenoxicam	0 (0,0)	1 (0,1)

Legend. [a] The number of exposures exceeds the number of pregnancies due to multiple exposure to study medication.
