# Supplementary material for: Fetal adverse effects following NSAID or metamizole exposure in the 2nd and 3rd trimester: an evaluation of the German Embryotox cohort
Source: BMC Pregnancy Childbirth. 2022 Aug 26;22:666. doi: 10.1186/s12884-022-04986-4 (PMC9413886; doi:10.1186/s12884-022-04986-4)
Supplement: Supplementary file 2 — Additional file 2: Table S2. Neonatal characteristics. [file 12884_2022_4986_MOESM2_ESM.pdf]

Table S2. Neonatal characteristics.

Cohorts	NSAID<br>n, 1098	Comparison<br>n, 1133
**GW at birth, n**	1095	1127
GW, median (IQR)	39 (38-40.1)	39.43 (38.1-40.3)
**Preterm birth, n**	1095	1127
Preterm (<GW 37), n (%)	162 (14.8)	117 (10.4)
Term, n (%)	933 (85.2)	1010 (89.6)
**Child's sex, n**	1091	1130
Female, n (%)	547 (50.1)	564 (49.9)
Male, n (%)	544 (49.9)	566 (50.1)
**Weight, n**	1090	1125
Median in g (IQR)	3300 (2940-3645)	3370 (3020-3710)
**Length, n**	1076	1115
Median in cm (IQR)	51 (49-53)	51 (49-53)
**Head circumference, n**	1035	1054
Median in cm (IQR)	35 (33.5-36)	35 (34-36)

Legend. The absolute number of parameters differs due to missing values. GW, gestational week; IQR, interquartile range; n, number of neonates with available information.
