# Supplementary material for: Fetal adverse effects following NSAID or metamizole exposure in the 2nd and 3rd trimester: an evaluation of the German Embryotox cohort
Source: BMC Pregnancy Childbirth. 2022 Aug 26;22:666. doi: 10.1186/s12884-022-04986-4 (PMC9413886; doi:10.1186/s12884-022-04986-4)
Supplement: Supplementary file 3 — Additional file 3: Table S3. Exposure intervals of study medication (a) and assignment of exposure to trimesters (b). [file 12884_2022_4986_MOESM3_ESM.pdf]

Table S3. Exposure intervals of study medication (a) and assignment of exposure to trimesters (b)

**a) Duration of exposure to NSAID and/or metamizole**	**Study cohort (n, 1092), exposure 2nd and/or 3rd trimester, n (%)**
**≤7 days**	366 (33,5)
**8-14 days**	84 (7,7)
**15-28 days**	78 (7,1)
**>28 days**	185 (16,9)
**Not further specified**	379 (34,7)
**Median (IQA) (min-max)**	7 (2-30) (1-280)

**b) Exposure to NSAID and/or metamizole per trimester**	**Study cohort (n, 1092), n (%)**
**2nd trimester only**	609 (55.8)
**3rd trimester only**	150 (13.7)
**2nd and 3rd trimester**	295 (27.0)
**2nd or 3rd trimester (not further assignable)**	38 (3.5)

Legend. n, number of cases; IQR, interquartile range. NSAID, non-steroidal anti-inflammatory drugs.
