# Supplementary material for: Fetal adverse effects following NSAID or metamizole exposure in the 2nd and 3rd trimester: an evaluation of the German Embryotox cohort
Source: BMC Pregnancy Childbirth. 2022 Aug 26;22:666. doi: 10.1186/s12884-022-04986-4 (PMC9413886; doi:10.1186/s12884-022-04986-4)
Supplement: Supplementary file 4 — Additional file 4: Table S4. Multiple exposure to study medication. [file 12884_2022_4986_MOESM4_ESM.pdf]

Table S4. Multiple exposure to study medication.

Number of substances	Study cohort, exposure: 2nd and/or 3rd trimester, n (%)	Comparison cohort, exposure: 1st trimester, n (%)
	**Pregnancies (n, 1092)**	**Pregnancies (n, 1154)**
1	960 (87.9)	947 (82.1)
2	118 (10.8)	165 (14.3)
≥3	14 (1.3)	42 (3.6)
**Mean value**	1.14	1.44
