# Supplementary material for: Fetal adverse effects following NSAID or metamizole exposure in the 2nd and 3rd trimester: an evaluation of the German Embryotox cohort
Source: BMC Pregnancy Childbirth. 2022 Aug 26;22:666. doi: 10.1186/s12884-022-04986-4 (PMC9413886; doi:10.1186/s12884-022-04986-4)
Supplement: Supplementary file 5 — Additional file 5: Table S5. Summary of cases with 2nd/3rd trimester study medication and defined study endpoints. [file 12884_2022_4986_MOESM5_ESM.pdf]

Table S5. Summary of cases with 2nd/3rd trimester study medication and defined study endpoints.

Case	GW at call	Study medication	Exposure study medication From GW	To GW	Dose (mg/d)	Indication for medication	Study endpoint and GW at diagnosis	GW at birth	Birth weight (percentile), sex	Additional remarks on pregnancy course, delivery and outcome of the neonate/infant	WHO-UMC causality assessment [a]
	Constriction of ductus arteriosus										
**1**	7+5	Diclofenac	33+3	33+4	75-150	pain	GW 33+4: constriction of ductus arteriosus and tricuspid insufficiency, live birth	36+0	50.-75. p., male	GW 34+6: mild tricuspid insufficiency, mild cardiomegaly, GW 35+3 normal fetal heart function; normal heart function after birth	certain
**2**	7+1	Acetyl salicylic acid	34+6	34+6	10000	attempted suicide	GW 34+6: increased diastolic flow in ductus arteriosus, live birth	39+4	10.-25. p., n/a	GW 35+0 normal diastolic flow; normal heart function after birth	probable
**3**	31+6	Ibuprofen	0	32+0	400-600 (3x/ week)	migraine	constriction of ductus arteriosus, heart valve insufficiency, GW unknown, live birth	41+1	25.-50. p., female	normal heart function after birth	probable
	Constriction of ductus arteriosus and oligohydramnios										
**4**	18+3	Diclofenac	0	27	150	rheumatoid arthritis	GW 22: amniotic fluid volume decreased; GW 26+5: Oligohydramnios and constriction of ductus arteriosus, live birth	35+1	3.-10. p., male	GW 34: normalisation of amniotic fluid volume and flow in ductus arteriosus; normal heart function after birth	constriction of ductus arteriosus: certain; oligohydramnios: certain
		Ibuprofen	16	20+5	600	rheumatoid arthritis					
**5**	26+6	Diclofenac	0	39	150-225	rheumatoid arthritis	premature closure of ductus arteriosus and amniotic fluid level decreased, GW unknown, live birth	39+0	50.-75. p., male	postnatal ultrasound 2 days after birth: ventricular hypertrophy, tricuspid insufficiency °I	constriction of ductus arteriosus: probable; oligohydramnios: possible
	Oligohydramnios										
**6**	13+1	Diclofenac	22+1	27+6	150	psoriatic arthritis	anhydramnios in GW 25, live birth	38+5	50.-75. p., male	treated with amnioinfusion, then oligohydramnios in GW 27, later normal amniotic fluid levels until birth	probable
**7**	11+6	Ibuprofen	18+2	18+4	1200	back pain	oligohydramnios in GW 20, live birth	38+0	50.-75. p., male	no further information	possible
**8**		Ibuprofen	0	26	400-800	pain in extremity	oligohydramnios in GW 21 (AFI 5.1), live birth	37+4	25.-50. p., female	control ultrasound (GW unknown): AFI 8	possible
**9**	10+6	Diclofenac	5+5	22+6	n/a	Bechterew's disease	oligohydramnios in GW 22+6, live birth	40+1	3.-10. p., female	later normalisation of amniotic fluid level, GW unknown	probable
		Metamizole	17+1	17+1	n/a	Bechterew's disease					

Case	GW at call	Study medication	Exposure study medication		Dose (mg/d)	Indication for medication	Study endpoint and GW at diagnosis	GW at birth	Birth weight (percentile), sex	Additional remarks on pregnancy course, delivery and outcome of the neonate/infant	WHO-UMC causality assessment [a]
			From GW	To GW							
**10**	12+2	Ibuprofen	0	3+0		headache	oligohydramnios in GW 23+1, live birth	38+1	25.-50. p., female	no further information	possible
		Ibuprofen	22+0	23+0	800 mg/d (5 days in total)	pain					
**11**	6+2	Ibuprofen	27	28	n/a	n/a	oligohydramnios, GW unknown, live birth	29+2	3.-10. p., female	IUGR	unclassifiable
**12**	8+0	Ibuprofen	0	30+2	600-1200 (when needed)	rheumatoid arthritis	Fetus 1: oligohydramnios, Fetus 2: oligohydramnios, IUGR, GW unknown, live birth	30+2	Fetus 1: 3.-10. p., female, Fetus 2: <3. p., female	discordant growth, placental insufficiency	possible
**13**	6+6	Ibuprofen	4+1	33+6	300-1200	Bechterew's disease	anhydramnios GW 30+2, induced abortion	33+6	<3. p., male	ultrasound GW 22: multiple abnormalities, suspected skeletal dysplasia; ultrasound GW 30: IUGR, anhydramnios, umbilical doppler abnormal; induced abortion in GW 34; pathology report: chronic placental insufficiency, fetal autopsy was not performed	oligohydramnios: possible
**14**	21	Ibuprofen	0	16	600 (when needed)	headache	oligohydramnios in GW 31+1, live birth	42+0	50.-75. p., male	no further information available	unlikely
**15**	11+2	Indometacin	31+1	31+3	150	shortened cervix	reduced amniotic fluid volume in GW 32, live birth	38+6	75.-90. p., female	no further information available	possible
**16**	34+4	Ibuprofen	23	24	2400	fever	oligohydramnios in GW 34+3, live birth	34+3	<3. p., male	IUGR, chronic placental insufficiency	unclassifiable
**17**	5+3	Ibuprofen	20	27	600-2400 (2x/week)	migraine	amniotic fluid volume decreased in GW 35+2, live birth	38+3	50.-75. p., female	no further information available	unlikely
**18**	15+0	Metamizole	13	35+5	n/a (when needed)	pain	oligohydramnios, GW week unknown (amniotic fluid volume might have been decreased throughout pregnancy), live birth	35+5	25.-50. p., male	no further information available	possible
**19**	17+5	Metamizole	13	37+4	n/a (when needed)	pain	oligohydramnios, GW unknown, live birth	37+4	10.-25. p., female	no further information available	unclassifiable
**20**	26+4	Diclofenac	0	28	75	rheumatoid arthritis	amniotic fluid volume decreased, GW unknown, live birth	37+6	75.-90. p., female	no further information available	unclassifiable

Case	GW at call	Study medication	Exposure study medication		Dose (mg/d)	Indication for medication	Study endpoint and GW at diagnosis	GW at birth	Birth weight (percentile), sex	Additional remarks on pregnancy course, delivery and outcome of the neonate/infant	WHO-UMC causality assessment [a]
			From GW	To GW							
**21**	24+6	Diclofenac	0	19+4	100mg/d (when needed)	rheumatoid arthritis	amniotic fluid volume decreased from GW 38, live birth	40+3	3.-10. p., male	IUGR	unlikely
**22**	5+5	Ibuprofen	0	26+6	400 (when needed)	headache	amniotic fluid volume decreased in GW 38 (in comparison to previous ultrasound examinations), live birth	38+0	<3. p., male	no further information available	unlikely
**23**	9+5	Ibuprofen	15	16	n/a	pain	oligohydramnios, GW unknown, live birth	38+3	50.-75. p., female	indication for labour induction	unlikely
**24**	5+3	Ibuprofen	0	28	n/a (when needed)	pain	oligohydramnios, GW unknown (probably amniotic fluid volume decreased throughout pregnancy), live birth	38+4	50.-75. p., male	no further information available	unclassifiable
**25**	5+1	Ibuprofen	8+5	16	300 (3 days in total)	migraine	oligohydramnios in GW 38+5, live birth	38+5	3.-10. p., female	GW 38+5: IUGR, indication for labour induction	unlikely
**26**	17+5	Ibuprofen	16	28	400 (4 days in total)	migraine	amniotic fluid volume decreased, GW unknown, live birth	38+5	3.-10. p., male	pre-eclampsia, GW unknown	unclassifiable
**27**	10+1	Ibuprofen	17+6	19+1	200-400 (2 days in total)	headache	oligohydramnios at term, live birth	39+3	3.-10. p., male	indication for labour induction	unlikely
**28**	11+5	Metamizole	15	15+1	1000	fever	amniotic fluid volume decreased, GW unknown, live birth	39+3	3.-10. p., male	no further information available	unclassifiable
**29**	13	Ibuprofen	24+5	24+5	600	rib pain	amniotic fluid volume decreased, GW unknown, live birth	39+3	75.-90. p., female	no further information available	unclassifiable
**30**	25+5	Ibuprofen	0	39+4	1200	fibromyalgia	oligohydramnios (probably around term), live birth	39+4	3.-10. p., female	suspected placental insufficiency	possible
**31**	19+4	Ibuprofen	0	33	600-800 (3-4x/month)	migraine	oligohydramnios at term, live birth	40+0	3.-10. p., female	indication for labour induction	unlikely
**32**	5+3	Ibuprofen	0	28	600 (1x/week)	migraine	oligohydramnios at term, live birth	40+1	3.-10. p., male	indication for labour induction	unlikely
**33**	33+3	Ibuprofen	0	28	n/a (when needed)	tension headache	oligohydramnios at term, live birth	40+1	10.-25. p., male	IUGR, placental insufficiency	unlikely

Case	GW at call	Study medication	Exposure study medication		Dose (mg/d)	Indication for medication	Study endpoint and GW at diagnosis	GW at birth	Birth weight (percentile), sex	Additional remarks on pregnancy course, delivery and outcome of the neonate/infant	WHO-UMC causality assessment [a]
			From GW	To GW							
**34**	32+6	Naproxen	0	32+2	1000	rheumatoid arthritis	oligohydramnios at term, live birth	40+2	10.-25. p., female	indication for labour induction	unlikely
**35**	32+4	Ibuprofen	5	26+3	400 (when needed)	migraine	oligohydramnios at term, live birth	40+5	10.-25. p., male	indication for labour induction	unlikely
**36**	21+2	Ibuprofen	0	27	400-800 (2-3x/ month)	migraine	oligohydramnios at term, live birth	40+5	<3. p., female	indication for labour induction	unlikely
**37**	4+5	Ibuprofen	0	15+5	600-1200	rheumatoid arthritis	oligohydramnios, GW unknown (probably around EDOB), live birth	40+6	50.-75. p., male	no further information available	unlikely
**38**	21+3	Indometacin	18+1	25+1	100-200 (13 days in total)	premature labour	oligohydramnios at term, live birth	41+0	50.-75. p., male	indication for labour induction	unlikely
**39**	7	Diclofenac	14	25	75 (2 days in total)	Bechterew's disease	oligohydramnios at term, live birth	41+0	50.-75. p., female	fetal tachycardia, indication for labour induction	unlikely
**40**	8+4	Ibuprofen	22+6	23	600	back pain	oligohydramnios in GW 42, live birth	41+5	50.-75. p., female	no further information available	unlikely
	Oligohydramnios AND tricuspid insufficiency or PDA**										
**41**	5+3	Ibuprofen	8	16	n/a, 2 tablets in total	pain	decreased amniotic fluid volume, GW unknown, mild tricuspid insufficiency 7 days after birth, live birth	38+4	50.-75. p., male	ultrasound 13 weeks after birth: normal heart function	unclassifiable
**42**	23+4	Diclofenac	23+3	23+3	150	bone pain	amniotic fluid volume decreased in the 2nd trimester, GW unknown; PDA in preterm born infant, live birth	31+5	90.-97. p., female	maternal haematological malignancy, elective caesarean section before beginning of treatment, spontaneous closure of PDA two days after birth	possible
		Ibuprofen	n/a	n/a	n/a	bone pain					
	Oligohydramnios AND abortion or stillbirth**										
**43**	24+4	Metamizole	23+1	24+4	1500	pain	anhydramnios in 2nd trimester, GW unknown, intrauterine death	26+3	<3. p., n/a	Crohn's disease, exacerbation in pregnancy, colectomy in GW 24, severe placental insufficiency and IUGR	oligohydramnios: possible; intrauterine death: unlikely
**44**	21+1	Ibuprofen	38+6	39	400	accidental exposure to product	amniotic fluid volume decreased in GW 40+0; intrauterine death in GW 40+6	40+6	25.-50. p., male	ultrasound in GW 40+0: AFI 3.7 cm; in GW 40+1: AFI 7.1cm; umbilical cord knot, immaturity of the placenta	oligohydramnios: possible; intrauterine death: unlikely

Case	GW at call	Study medication	Exposure study medication From GW	To GW	Dose (mg/d)	Indication for medication	Study endpoint and GW at diagnosis	GW at birth	Birth weight (percentile), sex	Additional remarks on pregnancy course, delivery and outcome of the neonate/infant	WHO-UMC causality assessment [a]
	Spontaneous abortion / stillbirth										
**45**	9+0	Ibuprofen	8+2	15	400 (when needed)	migraine	intrauterine death	15	n/a	Turner's syndrome	unlikely
**46**	8+3	Ibuprofen	0	15+4	400 (when needed)	migraine	intrauterine death	15+4	n/a	Trisomy 21, hydrops fetalis	unlikely
**47**	11+5	Indometacin	0	15	50 (every two weeks)	migraine	missed abortion	16+4	n/a	chromosomal abnormality (balanced Robertsonian translocation)	unlikely
**48**	14+6	Metamizole	14+5	15+3	5250	pain	late spontaneous abortion	18	n/a	haematological malignancy in pregnancy, chemotherapy, neutropenic sepsis	unlikely
		Ibuprofen	15+3	15+5	1200	pain					
**49**	13+0	Ibuprofen	12+1	15+4	1200-2000	pain	late spontaneous abortion	19+2	n/a	maternal sepsis, HELLP-syndrome, thrombosis	unlikely
**50**	4+2	Ibuprofen	0	19+1	n/a (1x/week)	pain	intrauterine death	19+3	n/a, male	pathology report: retroplacental haematoma	unlikely
**51**	7+0	Ibuprofen	8+6	20+5	800 (when needed)	migraine	intrauterine death	20+5	n/a, male	trisomy 1; multiple cardiac, urogenital, and cerebral malformations	unlikely
**52**	4+1	Ibuprofen	4+1	21	600-1200	back pain	late spontaneous abortion	21	10.-25. p., male	GW 6: parvovirus B19 infection, GW 20: cervical incompetence, GW 21: cerclage, amniotic infection	unlikely
**53**	14+3	Ibuprofen	19+4	21+1	400-800	migraine	late spontaneous abortion	21+4	n/a, male	severe purulent chorioamnionitis, premature separation of placenta	unlikely
**54**	25+5	Ibuprofen	0	26+3	n/a (1x/week)	headache	intrauterine death	26+3	n/a, female	severe preeclampsia	unlikely
**55**	5+3	Naproxen	0	27	500	rheumatic disorder	intrauterine death	36+1	50.-75. p., female	severe gestational diabetes; low weight placenta, disorder of circulation and maturation	unlikely
**56**	8+1	Ibuprofen	12	27	400-600 (when needed)	migraine	intrauterine death	36+2	<3. p., male	placental insufficiency, tight umbilical cord (2x), limb hypoplasia congenital, suspected trisomy 21	unlikely
	PDA / tricuspid insufficiency / right ventricular load / PPHT										
**57**	4+6	Ibuprofen	4+2	19	n/a, 2x in total	n/a	tricuspid insufficiency, ultrasound three days after birth, live birth	40+2	25.-50. p., female	maternal toxoplasmosis in GW 9, postnatal ultrasound: PFO, right-left-shunt, congenital heart disease	-
**58**	18+5	Ibuprofen	15+2	15+5	800	suspected myocarditis	twin 1: tricuspid insufficiency, live birth; twin	37+1	twin 1: 3.-10. p., female,	twin 1: small PFO, mild pyelectasis; twin 2: VSD, small PFO, hexadactyly left hand	-

Case	GW at call	Study medication	Exposure study medication<br>From GW	 To GW	Dose (mg/d)	Indication for medication	Study endpoint and GW at diagnosis	GW at birth	Birth weight (percentile), sex	Additional remarks on pregnancy course, delivery and outcome of the neonate/infant	WHO-UMC causality assessment [a]
							2: PDA in term born infant, live birth		twin 2: 10.-25. p., male		
**59**	4+6	Ibuprofen	0	20	n/a, daily intake	juvenile chronic arthritis	PDA in term born infant, PPHT, live birth	40+2	3.-10. p., male	postnatal examination: incomplete right bundle branch block, PFO; MRI: congenital cystic adenoid malformation of the lung	-
**60**	33+6	Metamizole	31+6	32+5	n/a, 2x	intercostal neuralgia	PDA in term born infant, live birth	38+4	<97. p., female	spontaneous closure of PDA after 4 weeks; PFO: after 22 months: spontaneous closure	-
**61**	32+4	Diclofenac	37+4	40+3	n/a, when needed	pain	PDA in term born infant, live birth	40+4	>97. p., female	postnatal ultrasound: VSD, PFO; no further information available	-
		Ibuprofen	37+4	40+3	n/a, when needed	pain					
**62**	8+6	Ibuprofen	24+3	32+5	n/a	neuralgia trigeminal	PDA in preterm born infant, live birth	32+6	25.-50. p., female	prenatal ultrasound: polyhydramnios, gestational diabetes, preeclampsia; postnatal ultrasound: esophagotracheal fistula Vogt IIIb, PFO, small PDA still haemodynamically relevant after 3 weeks, no further information	-
**63**	11+6	Ibuprofen	8+5	23+0	400-2400	rheumatoid arthritis	PDA in preterm born infant, live birth	28+1	10.-25. p., female	premature, preterm rupture of membranes and AIS; PDA: unsuccessful closure attempt with ibuprofen (2x), additional heart insufficiency, surgical closure 17 days after birth	-
**64**	5+5	Etoricoxib	0	26+0	120	pain	PDA in preterm born infant, live birth	28+0	50.-75. P., female	small PFO or VSD, no further information	-
**65**	16+1	Ibuprofen	6+1	18+0	n/a	pain	PDA in preterm born infant, PPHT, tricuspid insufficiency, neonatal death 20h after birth	28+5	75.-90. p., female	anhydramnios after premature preterm rupture of membranes, chorioamnionitis, lung hypoplasia	-
**66**	5+2	Metamizole	13	32+2	4000	fever	PDA in preterm born infant, live birth	32+2	75.-90. p., female	umbilical cord knot, PDA closed 4 days after birth	-
		Ibuprofen	0	13	n/a (when needed)	lupus erythema-tosus					
**67**	20+6	Ibuprofen	13+0	26+6	400	pain	PDA in preterm born infant, live birth	36+6	>97. p., male	preeclampsia, pre-existent maternal diabetes; spontaneous closure of PDA, no further information	-
**68**	8+3	Diclofenac	0	28+0	50	juvenile rheumatoid arthritis	PDA in preterm born infant, live birth	29+0	3.-10. p., female	prenatal ultrasound: IUGR, placental insufficiency; postnatal ultrasound: PFO; PDA: successful closure 3 days after birth after treatment with indometacin	-

Case	GW at call	Study medication	Exposure study medication From GW	To GW	Dose (mg/d)	Indication for medication	Study endpoint and GW at diagnosis	GW at birth	Birth weight (percentile), sex	Additional remarks on pregnancy course, delivery and outcome of the neonate/infant	WHO-UMC causality assessment [a]
**69**	8+5	Metamizole	23+5	25	n/a	pain	PDA in preterm born infant, live birth	25+6	25.-50. p., female	mother: oncologic disease; infant: PDA treated with ibuprofen 9 days after birth and indometacin from day 13 after birth, at day 31 PDA still slightly open, closed PDA 71 days after birth; ASD	-
**70**	7+1	Ibuprofen	0	29+5	n/a (when needed)	pain	PDA in preterm born infant, live birth	29+6	3.-10. p., male	spontaneous closure of PDA, no further information	-
**71**	8+4	Indometacin	29+3	29+3	n/a	tocolysis	PDA in preterm born infant, live birth	29+5	75.-90. p., male	PDA still open three months after birth, closed 6 months after birth, VSD (spontaneous closure), PFO	-
		Ibuprofen	4+5	5+0	1200	swine influenza					
	Renal disorders										
**72**	13+5	Ibuprofen	0	22+0	400-800 mg/d (when needed)	migraine	3 months after birth: unilateral renal hypoplasia, enlarged other kidney, live birth	39+2	50.-75. p., male	infant in good general condition, control examination planned 6 months later	-

Legend. Study cases with multiple study endpoints are only listed once. [a] World Health Organisation – Uppsala Monitoring Centre; causality assessment was only performed for prenatal endpoints. Co-medication is not listed but was considered. AIS, amniotic infection syndrome; AFI, amniotic fluid index; d, days; GW, gestational week; IUGR, intrauterine growth retardation; mg, milligram; n/a, not available; PDA, patent ductus arteriosus; PFO, patent foramen ovale; PPHT, primary pulmonary hypertension of the newborn; VSD, ventricular septal defect.
